# Impact of changing pre‐exposure prophylaxis regimens on retention among men who have sex with men in Hanoi, Vietnam (2020−2023): a cohort study

**DOI:** 10.1002/jia2.26478

**Published:** 2025-07-02

**Authors:** Naira Setrakian, Hao Thi Minh Bui, Paul C. Adamson, Thai N. Hoang, Pamina M. Gorbach, Le Minh Giang

**Affiliations:** ^1^ Department of Epidemiology UCLA Fielding School of Public Health Los Angeles California USA; ^2^ Hanoi Medical University Hanoi Vietnam; ^3^ Division of Infectious Diseases David Geffen School of Medicine at UCLA Los Angeles California USA; ^4^ Division of Global HIV and Tuberculosis (DGHT) U.S. Centers for Disease Control and Prevention Hanoi Vietnam

**Keywords:** pre‐exposure prophylaxis; HIV prevention, men who have sex with men, Vietnam, epidemiology, retention

## Abstract

**Introduction:**

We examined the association of pre‐exposure prophylaxis (PrEP) programme retention with the use of daily, event‐driven (ED) or regimen switching reported during follow‐up at any point prior to discontinuation among men who have sex with men (MSM) in Hanoi, Vietnam.

**Methods:**

Between April 2020 and February 2023, we collected data from PrEP clients at Hanoi Medical University Sexual Health Promotion clinic who were prescribed either ED or daily PrEP at the initial visit; at subsequent visits, clients reported the regimen used since the prior visit. We defined three categories of PrEP use: ED‐PrEP exclusively, daily PrEP exclusively and switching regimens. The primary outcome was time to discontinuation in the PrEP programme during the study period, defined as missing a scheduled visit by > 30 days. We performed survival analysis using Kaplan−Meier curves.

**Results:**

In total, 2107 people were included: 61.1% (*n* = 1288) reported exclusive use of daily PrEP, 10.4% (*n* = 220) reported exclusive use of ED‐PrEP and 28.4% (*n* = 599) reported switching PrEP regimens. Among switchers, 29.40% (*n* = 176) switched more than once. Furthermore, 82.5% switched from daily to ED‐PrEP and 17.5% switched from ED to daily PrEP. The median time to discontinuation in the PrEP programme was 105 days (IQR: 52−182) among those reporting exclusive use of ED‐PrEP, 104 days (IQR: 56−274) among those reporting exclusive use of daily PrEP and 163 days (IQR: 101−308) among those who switched. Among switchers, those who switched more than once had a median time to discontinuation in the PrEP programme of 231 days (IQR: 137−380) in comparison to 133 days (IQR: 90−274) for those who switched once.

**Conclusions:**

We provide real‐world data from MSM in an HIV PrEP programme in Vietnam that those who switched had longer periods of retention during the study period. Our findings suggest that offering flexible PrEP regimen options may improve engagement and long‐term adherence among this population.

## 1 INTRODUCTION

Oral pre‐exposure prophylaxis (PrEP) is a highly effective method for preventing HIV transmission, and the World Health Organization (WHO) recommends the use of PrEP for populations at substantial risk of acquiring HIV [1]. Daily PrEP, which involves taking one pill daily, was approved by the U.S. Food and Drug Administration (FDA) in 2012; since then, 144 countries globally have adopted WHO recommendations for oral PrEP in their national guidelines [2]. In 2019, the WHO updated its recommendations for PrEP to include event‐driven PrEP (ED‐PrEP), also known as on‐demand PrEP, as a prevention method for HIV transmission among men who have sex with men (MSM) [2]. ED‐PrEP involves taking two pills 2–24 hours before sex, followed by one pill 24 and 48 hours later, as opposed to daily PrEP, which is the conventional method. ED‐PrEP is a cost‐effective and convenient option for MSM, especially for individuals who engage in less frequent sex, can plan for sex in advance or prefer not to take daily PrEP [2]. With the emergence of ED‐PrEP, studies have sought to understand patterns of PrEP initiation and reasons for PrEP preferences among MSM [3, 4].

Evidence suggests that changes in individuals’ behaviours and circumstances—such as partnership status, substance use and sexual activity—lead to evolving needs around PrEP over the individual life course, which in turn influence patterns of use and engagement with PrEP programmes [5−7]. Perceptions of risk have also been shown to impact PrEP use, with individuals reporting low sexual activity, monogamous relationships or the end of a relationship as reasons for missing PrEP doses [8, 9]. While these factors are all known to change individual PrEP use patterns over time, there have been limited opportunities to quantify these patterns; particularly, how individuals switch between different PrEP regimens and the association this has with their engagement in PrEP care.

The prevalence of HIV among MSM in Vietnam continues to increase, despite low prevalence rates in the general population [10]. PrEP has been demonstrated to be effective in preventing HIV transmission among MSM in Vietnam [11]. Daily PrEP has been offered in Vietnam since 2019, with ED‐PrEP approved in 2020 [12]. Patterns of uptake, retention and switching between ED‐PrEP and daily PrEP regimens remain largely unknown across Vietnam.

Understanding the relationship between regimen switching and engagement with PrEP care can help identify the best strategies for optimizing PrEP care and ultimately reducing HIV transmission in resource‐limited settings. The objective of this study was to examine how regimen switching between daily and ED‐PrEP was associated with retention within a real‐world PrEP programme for MSM in Vietnam.

## 2 METHODS

This was a secondary analysis of HIV PrEP data from the Sexual Health Promotion (SHP) clinic of Hanoi Medical University (HMU). The SHP clinic provides HIV PrEP as the centre of a comprehensive HIV prevention model, which includes screening and management of treatment for sexually transmitted infections, treatment for substance use disorder and mental healthcare for people at risk for HIV acquisition. PrEP dispensing at the SHP clinic was implemented in 2019 during the rollout of the national PrEP programme. SHP is currently the largest PrEP clinic in Hanoi, and as of June 2023, has provided 4425 clients with PrEP, allowing clients to choose between daily or ED regimens. Between 1 April 2020 and 21 February 2023, routinely collected programmatic data from those who initiated HIV PrEP at the SHP clinic were assessed. 21 February 2023 was selected as an arbitrary cutoff to facilitate data analysis. Analysis was restricted to clients who were male at birth, identified as male, reported having sex with only males or both males and females, and returned for follow‐up.

At PrEP programme enrolment, clients were provided with PrEP counselling and prescribed either ED‐PrEP or daily PrEP. Clients were first administered a series of demographic and sexual behavioural questions as part of a risk behaviours assessment. At the PrEP initiation or reinitiation visit, providers presented information on both PrEP regimens to clients and conducted a risk assessment based on sexual practices. Results of a risk assessment were used to guide a discussion with the client, allowing them to decide on the most appropriate regimen at the time of PrEP initiation. Clients were encouraged to contact the clinic staff for any assistance, including guidance on PrEP regimen adherence and switching. HIV PrEP medication (emtricitabine 200 mg/tenofovir disoproxil fumarate 300 mg) was provided at the clinic free of charge. Per national guidelines, clients were asked to return 30 days following their initial in‐person visit, 60 days after their first revisit, and subsequently every 90 days thereafter [13]. A follow‐up appointment date was set up after each visit. Clients were expected to return within 30 days of their set appointment date. At each follow‐up visit, clients reported the type of PrEP used since their prior visit following a standard form filled out by clinic staff. Clinic staff also addressed all questions and concerns from clients about PrEP use and regimens at every visit. Clients were grouped by the type of PrEP they used. They were provided with the option to choose their PrEP type at each visit.

For analysis, clients were grouped into the following categories: (1) chose ED‐PrEP at baseline and exclusively reported ED‐PrEP throughout follow‐up; (2) chose daily PrEP at baseline and exclusively reported daily PrEP throughout follow‐up; and (3) switched PrEP regimen type at any point during follow‐up. To determine client grouping, the type of PrEP received at baseline was compared to the type of PrEP reported during follow‐up. Those who switched to a different regimen were categorized as those who switched. Those who switched were then sub‐categorized into those who switched more than once and those who switched only once.

The primary outcome of interest was time to discontinuation in the PrEP programme, defined as the event in which clients did not return for a visit within 30 days of their next appointment date. A client was marked as “discontinued” on the date of their last visit. For the purpose of this analysis, we did not evaluate subsequent follow‐up data for clients who returned after their first discontinuation event. Importantly, retention refers only to retention in the PrEP programme at the SHP clinic. Clients who had appointment dates scheduled after 20 January 2023, which was 30 days prior to the data cutoff date, were evaluated as retained clients. In addition, clients who had an initial appointment on or after 23 December 2022, which was 60 days prior to the data cutoff date, were excluded from the analysis to ensure that all clients included had sufficient time for follow‐up. Clients who only had an initial visit were also excluded from the analysis to ensure comparable person‐time between those reporting exclusive use of ED‐PrEP or daily PrEP and those who switched regimens. This criterion was necessary because categorizing a client as a switcher required data from more than one visit.

The exposures of interest, all measured once at baseline, were: age, sex at birth of sex partners, number of sex partners in the prior 6 months, self‐reported condomless anal sex with someone perceived to increase their risk for HIV acquisition, sexualized drug use (defined as using drugs for the purposes of sex) in the prior 6 months and having a spouse/partner living with HIV. Univariate analyses were used to describe the baseline characteristics and behaviours of clients who received PrEP from the clinic. Socio‐demographic and sexual behavioural factors were extracted from the client medical records. The mean and median overall follow‐up time were calculated for each group using months since the initial visit.

Survival analyses using Kaplan−Meier curves were performed to assess the time‐to‐event patterns of PrEP retention within those with exclusive ED‐PrEP use, those with exclusive daily PrEP use and those who switched regimens. Time‐to‐event was defined as days from the first visit during which PrEP was initiated at the clinic until the discontinuation in the PrEP programme. Median time to discontinuation in the PrEP programme and associated interquartile ranges (IQRs) were calculated for each group. Kaplan−Meier survival curves were also generated for one‐time switchers and multi‐switchers. Log‐rank tests were used to test for significant differences between the survival curves.

Simple Cox proportional hazard models of time to discontinuation in the PrEP programme by baseline characteristics and behaviours, including age, sex of sex partners, number of sex partners in the prior 6 months, condomless anal sex with someone perceived to increase their risk for HIV acquisition, sexualized drug use in the prior 6 months and spouses/partners living with HIV, were calculated for each group to identify factors associated with retention. For all Cox proportional hazard models, we report hazard ratios (HRs), 95% confidence intervals (95% CIs) and *p*‐values. All statistical analyses were conducted using R, version 4.2.3, using packages dplyr, survival, survminer and ggplot2 [14−17].

The study was approved by the Institutional Review Board of Hanoi Medical University (#769/HMU‐IRB) and the Institutional Review Board at the University of California, Los Angeles (IRB#23‐001777). A waiver of informed consent was obtained due to the minimal‐risk nature of the research and the use of de‐identified data. This activity was reviewed by the CDC, deemed not research, and was conducted consistent with applicable federal law and CDC policy.

## 3 RESULTS

Among the 4034 clients who initiated PrEP during the study period, 2107 clients met the inclusion criteria and were included in the analysis (Table 1). Of the initial 4034 clients, 25.7% (*n* = 1037) did not return after the first visit. Additionally, 2.2% (*n* = 90) did not currently identify as a male, and less than 2.0% (*n* = 79) were born female at birth. Furthermore, 23.2% (*n* = 936) did not have data on sex of sex partner, 2.6% (*n* = 104) reported having sex exclusively with females and 0.2% (*n* = 10) reported never having had sex before. These individuals were all excluded from the analysis. It is important to note that a portion of individuals met multiple exclusion criteria.

**Table 1:** Baseline demographic and behavioural characteristics of clients (*N* = 2107) receiving PrEP from the Sexual Health Promotion clinic in Hanoi, Vietnam between April 2020 and February 2023, stratified by regimen type

	Total		ED‐PrEP		Daily PrEP		Switchers		
	*N*	%	*n*	%	*n*	%	*n*	%	*p*‐value
*n*	2107		220	10.4%	1288	61.1%	599	28.4%	
Age, years, median and IQR	24.2 (21.1−28.1)		23.1 (20.4−26.8)		24.64 (21.4−28.8)		23.7 (20.8−26.9)		
Overall follow‐up time in months, median and IQR	6.5 (3.18−15.0)		4.2 (1.9−7.7)		6.4 (3.0−14.8)		8.1 (4.0−18.6)		
Sex of sex partner									<0.01
Males	1715	81.1%	196	88.7%	1011	78.3%	508	84.4%	
Males and females	392	18.6%	24	10.9%	277	21.5%	91	15.2%	
Number of sex partners, prior 6 months									<0.01
1	562	26.7%	78	35.4%	323	25.1%	161	26.7%	
≥2	1434	68.1%	116	52.5%	914	71.0%	404	67.4%	
Condomless sex with people having high HIV‐related risks, prior 6 months									<0.01
Yes	1095	52.0%	83	37.6%	699	54.3%	313	52.0%	
No	1012	48.1%	137	62.3%	589	45.7%	286	47.7%	
Drug use for the purpose of sex, prior 6 months									0.08
Yes	209	9.9%	31	14.0%	125	9.7%	53	8.8%	
No	1898	90.1%	189	85.9%	1163	90.3%	546	91.2%	
Spouses/partners living with HIV									0.13
Yes	55	2.6%	3	1.4%	41	3.2%	11	1.8%	
No	2052	97.4%	217	98.6%	1247	96.8%	588	98.2%	

*Note*: *p*‐values calculated using chi‐squared tests and Fisher's exact tests and represent significant differences in prevalence of baseline characteristics across groups.

Abbreviations: ED‐PrEP, event‐driven PrEP; IQR, interquartile range; PrEP, pre‐exposure prophylaxis.

Of the 2107 included in the analysis, 84.6% chose daily PrEP and 15.4% chose ED‐PrEP at the first visit. Overall, 10.4% (*n* = 220) of clients reported exclusive use of ED‐PrEP, 61.1% (*n* = 1288) reported exclusive use of daily PrEP and 28.4% (*n* = 599) were classified as switchers. Among switchers, 29.40% (*n* = 176) switched more than once. Furthermore, among the 599 switchers, 82.5% switched from daily to ED‐PrEP and 17.5% switched from ED to daily PrEP.

The median age was 24.2 (IQR: 21.1−28.1) years. The majority of all clients reported having sex with males only (81.1%). The prevalence of condomless sex in the prior 6 months was highest among those who reported exclusive use of daily PrEP (54.3%) and switchers (52.0%). Having two or more sex partners in the prior 6 months was most frequent among those reporting exclusive use of daily PrEP (71.0%) and switchers (67.4%). Using drugs for the purpose of sex in the prior 6 months was reported by 14.0% of those who reported exclusive use of ED‐PrEP, 9.7% of those who reported exclusive use of daily PrEP and 8.8% of switchers.

**Figure 1:**
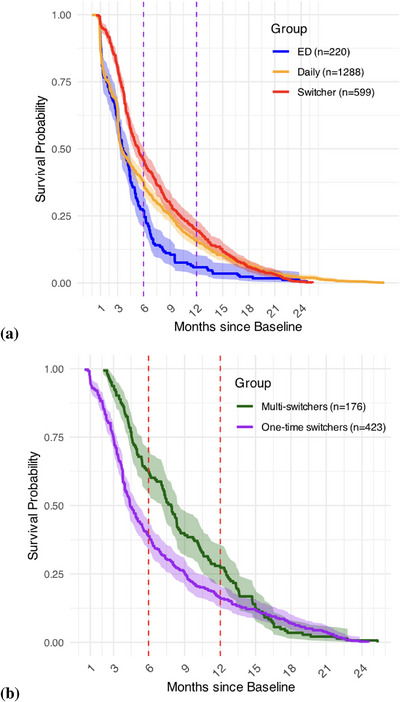
**Kaplan−Meier curves of time to discontinuation in the PrEP programme among people in a PrEP programme in Hanoi, Vietnam**. (a) Time to discontinuation in the PrEP programme comparing ED‐PrEP (median = 105 days, 52–182), daily PrEP (median = 104 days, IQR: 56–274) and switcher groups (median = 163, IQR: 101–308). A log‐rank test indicated a statistically significant difference between the three groups (*p* < 0.01). (b) Time to discontinuation in the PrEP programme comparing one‐time switchers (median = 133, IQR: 90–274) and multi‐switchers (median = 231 days, IQR: 137–380) (*p* < 0.01). A log‐rank test indicated a statistically significant difference between the two groups (*p* < 0.01). Abbreviations: ED‐PrEP, event‐driven PrEP; IQR, interquartile range; PrEP, pre‐exposure prophylaxis.

The median time to discontinuation in the PrEP programme for those who reported exclusive use of ED‐PrEP was 105 days (IQR: 52−182) compared to those who reported exclusive use of daily PrEP which was 104 days (56−274) (log‐rank test *p* < 0.01). The median time to discontinuation in the PrEP programme for switchers was 163 days (IQR: 101−308) and longer than those who reported exclusive use of ED‐PrEP and daily PrEP (log‐rank test *p* < 0.01) (Figure 1a). Among switchers, multi‐switchers had a median time to discontinuation in the PrEP programme of 231 days (IQR: 137−380) in comparison to 133 days (IQR: 90−274) for one‐time switchers (log‐rank test *p* < 0.01) (Figure 1b). Of the *n* = 481 switchers who discontinued, 67.6% reported daily PrEP use and 32.4% reported ED‐PrEP use at the last visit.

Among those who reported exclusive use of ED‐PrEP, no significant differences in HRs of discontinuation in the PrEP programme by any baseline characteristics were found (Table 2). Among both those who reported exclusive daily PrEP and switchers, the only factor associated with time to discontinuation in the PrEP programme was condomless anal sex with someone perceived to increase their risk for HIV acquisition (HR = 0.80, 95% CI [0.70, 0.92] and HR = 0.82, 95% CI [0.69, 0.99], respectively).

**Table 2:** Adjusted hazard ratios of discontinuation in the PrEP programme using Cox proportional hazard models by baseline characteristics and behaviours of clients (*N* = 2107) receiving PrEP from the Sexual Health Promotion clinic in Hanoi, Vietnam between April 2020 and February 2023, stratified by PrEP regimen type

	ED‐PrEP		Daily PrEP		Switchers	
	aHR	95% CI	aHR	95% CI	aHR	95% CI
Age (years)	1.02	0.98, 1.06	1.00	0.99, 1.01	0.99	0.97, 1.01
Sex of sex partner						
Males and females	−	−	−	−	−	−
Males	1.19	0.72, 1.98	1.11	0.95, 1.30	1.08	0.85, 1.38
Number of sex partners in prior 6 months						
1	−	−	−	−	−	−
≥2	1.01	0.73, 1.39	0.93	0.80, 1.09	1.02	0.83, 1.25
Condomless sex with people having high HIV‐related risks in prior 6 months						
No	−	−	−	−	−	−
Yes	0.84	0.62, 1.14	0.80^**^	0.70, 0.92	0.82^*^	
	0.69, 0.99					
Spouses/partners living with HIV						
No	−	−	−	−	−	−
Yes	1.50	0.37, 6.09	1.14	0.76, 1.73	0.69	0.34, 1.39

Abbreviations: aHR, adjusted hazard ratio; CI, confidence interval; ED‐PrEP, event‐driven PrEP; PrEP, pre‐exposure prophylaxis.

* *p*‐value <0.05.

** *p*‐value <0.01.

## 4 DISCUSSION

Our findings, based on real‐world data from an HIV PrEP programme in Hanoi, Vietnam, over a 3‐year period, provide important insights into PrEP use patterns among MSM and men who have sex with men and women in Vietnam. PrEP regimen switching was common, with more than a quarter of clients changing between daily and ED‐PrEP. While most clients initiated daily PrEP without switching, those who switched regimens at any point had longer overall retention compared to those who did not switch. Additionally, among those who switched, clients who switched more than once had a longer time to discontinuation than those who switched only once. These findings suggest that offering flexible PrEP options, including the anticipated introduction of long‐acting injectable PrEP in Vietnam, may improve engagement in this population, whose prevention needs fluctuate over time. Notably, approximately a quarter of clients discontinued PrEP after their first visit, demonstrating that PrEP use and retention often reflect evolving individual needs. These trends align with findings from PrEP programmes in other Southeast Asian countries [18, 19].

Understanding socio‐demographic and behavioural determinants of PrEP uptake and retention is critical for improving HIV prevention among MSM and men who have sex with men and women. Our study highlights distinct behavioural patterns among individuals who use PrEP, particularly regarding condomless sex and number of sexual partners. Clients reporting exclusive daily PrEP use and those who switched regimens were more likely to report condomless sex with partners perceived to increase their risk for HIV acquisition and to report multiple partners in the past 6 months, compared to exclusive ED‐PrEP users. These findings align with the idea that ED‐PrEP is more commonly used by those with less frequent sexual activity, whereas switching may be driven by changes in sexual behaviour and perceived risk of HIV acquisition [19, 20]. Additionally, among those with exclusive daily PrEP use and switchers, reporting condomless sex with partners perceived to increase their risk for HIV acquisition was associated with lower hazard rates of discontinuation. This pattern was not observed among those with exclusive ED‐PrEP use, suggesting that retention in PrEP care may be higher among those who perceive themselves at greater risk for acquiring HIV. Similar findings have been reported in U.S.‐based studies, where PrEP adherence was linked to having more partners, lower perceived importance of condoms and engaging in condomless sex with partners living with HIV [21, 22]. These trends suggest that PrEP retention is influenced by both behavioural factors and individual perceptions of HIV acquisition risk.

Our analysis was completed using real‐world programmatic data from the largest provider of PrEP services in Hanoi, Vietnam. As such, it is likely that clients attending the PrEP clinic are not representative of the general population of MSM in Hanoi, including hard‐to‐reach participants who are increasingly vulnerable to HIV, but do represent MSM in Hanoi seeking PrEP. Given that information regarding socio‐demographic characteristics and sexual behaviours are only collected at HIV PrEP initiation, we are unable to draw conclusions about the longitudinal effect of these factors on PrEP use. It is possible that individuals who remain in PrEP care continue to experience varying levels of sexual risk, whereas those who discontinue may have transitioned into periods of low or no‐risk exposure. Furthermore, this study was a retrospective analysis of programmatic data, limiting our ability to establish causal relationships between PrEP regimen choice and retention outcomes. Additionally, because this was a retrospective study, we relied on existing clinic records, which may not comprehensively capture changes in clients’ sexual behaviours, PrEP adherence or reasons for discontinuation over time. Behavioural data in this study were collected directly by medical personnel as part of routine medical care. While the clinic staff and clinicians are well‐trained in providing patient‐centred care, some behaviours might be under‐reported due to potential stigma and/or social desirability bias. In the future, providing clients with self‐administered questionnaires that allow for confidential reporting of sexual and drug use practices might result in less potential underreporting of these behaviours.

In order to account for immortal person‐time bias, we chose to exclude clients with only one visit. Clients who did not return for follow‐up after the first visit did not appear to differ by socio‐demographic or behavioural characteristics from those who did return. Including those with only one visit in the analysis caused a significant decrease in median survival time for those who reported ED‐PrEP and daily PrEP, since switchers, by definition, are required to have more than one visit to be categorized as a switcher. Given that the analysis focused on the retention of PrEP, it is important to note that clients may have returned to the clinic beyond the 60‐day window period of their next appointment. Within this population, 50% of those who reported exclusive ED‐PrEP use, 43% of those who reported exclusive daily PrEP use and 62% of switchers returned to the clinic after first discontinuation in the PrEP programme. The median time from first discontinuation to return was approximately 3 months for those with ED‐PrEP use and those who switched, whereas it was 2 months for those using daily PrEP. Although the 30‐day window may lead to an underestimation of retention among PrEP clients, this timeframe aligns with the national PrEP programme of Vietnam, which utilizes an electronic database system to track client data. According to this system, clients who miss their PrEP appointment by 30 days or more are marked as “stopped.” Furthermore, our analysis does not provide a precise measure of PrEP coverage due to limitations in the available clinic data, which only includes follow‐up visits. As a result, PrEP coverage—particularly for ED‐PrEP—may be underestimated if clients used their supplies appropriately but had infrequent exposures, allowing them to extend their supply without needing to return for scheduled visits. Thus, they may still be using PrEP between missed appointments. Additionally, our database does not track clients who may have switched to a different clinic for PrEP, meaning some individuals classified as “retained” in our analysis may actually be continuing PrEP care elsewhere. This could lead to an overestimation of retention within our clinic‐specific data.

Despite these limitations, the chosen variable for measuring PrEP retention serves as a valid proxy for PrEP coverage, given the prominence of the SHP clinic as the largest PrEP provider in Hanoi, Vietnam. The clinic's clients receive thorough guidance in their decision‐making process and regularly return for prescription pick‐ups. Moving forward, we recommend implementing a more comprehensive follow‐up system in which clients are contacted multiple times after missed appointments. Additionally, gathering more information on lapses in care through qualitative interviews could provide deeper insights into whether clients are seeking care elsewhere or discontinuing PrEP use altogether.

## 5 CONCLUSIONS

Switching between daily and ED‐PrEP was common in this large PrEP programme in Hanoi, Vietnam and was associated with longer retention in PrEP care. These findings suggest that offering flexible PrEP regimen options may improve engagement and long‐term adherence among MSM and men who have sex with men and women. Given the real‐world nature of our data, our results highlight the importance of accommodating clients’ changing prevention needs over time. To strengthen PrEP retention and effectiveness, future efforts should focus on identifying factors that influence PrEP use patterns through longitudinal research and qualitative interviews. Understanding clients’ motivations for switching regimens and addressing barriers to continued PrEP use can help optimize programmatic strategies. Additionally, expanding PrEP services to reach populations at higher risk of HIV acquisition, including those who are harder to engage in care, is essential for improving overall HIV prevention efforts.

## COMPETING INTERESTS

All authors declare no conflicts of interest.

## AUTHORS’ CONTRIBUTIONS

Concept and design: NS, PCA, HTMB and PMG. Acquisition, analysis or interpretation of data: NS. Drafting of the manuscript: NS, PCA, HTMB and PMG. Critical review of the manuscript for important intellectual content: NS, PCA, HTMB, TNH, PMG and LMG. Statistical analysis: NS. Obtained funding: PCA and HTMB. Administrative, technical or material support: HTMB. Supervision: PMG and LMG. All authors have read and approved the final manuscript.

## FUNDING

This research has been supported by the President's Emergency Plan for AIDS Relief (PEPFAR) through the Centers for Disease Control and Prevention (CDC) under the terms of the Award #GH002150. This work was also supported by the National Institutes of Health, Fogarty International Center (K01TW012170 to PCA). HTMB was supported by the Fogarty International Center and the Office of Disease Prevention (D43TW009343) and the University of California Global Health Institute (UCGHI).

## DISCLAIMER

The funders had no role in the data collection, analysis or interpretation, or the decision to submit to publication. The findings and conclusions in this report are those of the author(s) and do not necessarily represent the official position of the funding agencies.

## Data Availability

Data are not publicly available. De‐identified data and other supporting documents will be made available upon reasonable requests made to the corresponding and the last authors. Researchers interested in accessing data should submit a formal request outlining their research objectives and plan to ensure data security.
